# Comparative Treatment Failure Rates of Respiratory Fluoroquinolones or β-Lactam + Macrolide Versus β-Lactam Alone in the Treatment for Community-Acquired Pneumonia in Adult Outpatients: An Analysis of a Nationally Representative Claims Database: Erratum

**DOI:** 10.1097/01.md.0000473517.85992.17

**Published:** 2015-10-30

In the article “Comparative Treatment Failure Rates of Respiratory Fluoroquinolones or β-Lactam + Macrolide Versus β-Lactam Alone in the Treatment for Community-Acquired Pneumonia in Adult Outpatients: An Analysis of a Nationally Representative Claims Database”,^1^ which appeared in Volume 94, Issue 39 of *Medicine*, the term “fluoroquinolones” was originally misspelled in the abstract. The article has since been corrected online.
